# Eosinopenia as Predictor of Poor Outcome in Hospitalized COVID-19 Adult Patients from Waves 1 and 2 of 2020 Pandemic

**DOI:** 10.3390/microorganisms10122423

**Published:** 2022-12-07

**Authors:** Raphael Cauchois, Lea Pietri, Jean-Baptiste Dalmas, Marie Koubi, Thibaut Capron, Nadim Cassir, Nicola Potere, Ildo Polidoro, Rodolphe Jean, Pierre-André Jarrot, Baptiste Andre, Veronique Veit, Julien Carvelli, Vanessa Pauly, Pascal Chanez, Laurent Papazian, Gilles Kaplanski

**Affiliations:** 1 Hôpital de la Conception, Internal Medicine and Clinical Immunology Department, APHM, INSERM, INRAE, C2VN, Aix Marseille Université, 13005 Marseille, France; 2 Hôpital de la Conception, Endocrinology Department, APHM, Aix Marseille Université, 13005 Marseille, France; 3 Hôpital Nord, Clinique des Bronches, Allergies et Sommeil Department, APHM, Aix Marseille Université, 13005 Marseille, France; 4 IHU-Méditerranée Infection Department, IRD, AP-HM, MEPHI, Aix Marseille Université, 13005 Marseille, France; 5 Department of Innovative Technologies in Medicine and Dentistry, “G. d’Annunzio” University, 66100 Chieti, Italy; 6 Unit of Forensic Medicine, Local Health Authority of Pescara, 65100 Pescara, Italy; 7 Hôpital de la Timone, Internal Medicine Department, APHM, Aix Marseille Université, 13005 Marseille, France; 8 Hôpital de la Timone, Réanimation des Urgences Department, APHM, Aix Marseille Université, 13005 Marseille, France; 9 CEReSS-Health Service Research and Quality of Life Center, Department of Medical Information, Hôpital de la Conception, APHM, 13005 Marseille, France; 10 Hôpital Nord, Réanimation des Détresses Respiratoires et Infections Sévères Department, APHM, Aix Marseille Université, 13005 Marseille, France

**Keywords:** eosinopenia, COVID-19, prognosis, biomarker

## Abstract

During SARS-CoV-2 infection, eosinopenia may reflect a hyperactive immune response. In this study of hospitalized COVID-19 patients, we aimed to better understand the prognostic value of severe eosinopenia (absolute eosinophil count = 0 G/L) and decipher its underlying mechanisms. We retrospectively analyzed the records of COVID-19 patients hospitalized from March to June 2020 in three university hospitals in Marseille, France. We assessed the association between severe eosinopenia and a composite poor outcome in these patients, including the need for oxygen supplementation at >6 L/min, ICU admission, and in-hospital death. Among the 551 COVID-19 patients included in this study, severe eosinopenia was found in 228 (51%) of them on admission to hospital and was associated with a composite poor outcome using multivariate analysis (OR = 2.58; CI95 [1.77–3.75]; *p* < 0.0001). We found a significant association between the presence of severe eosinopenia on admission and the elevation in C-reactive protein, ferritin, IP-10, and suPAR. The histological findings in a series of 37 autopsies from patients who died from severe COVID-19 and presented with severe eosinopenia showed no pulmonary eosinophil trapping. Severe eosinopenia can be a reliable biomarker associated with a composite poor outcome in hospitalized COVID-19 adult patients. It may reflect the magnitude of immune hyperactivation during severe-to-critical COVID-19.

## 1. Introduction

The outbreak of the coronavirus disease 19 (COVID-19) caused by severe acute respiratory syndrome coronavirus 2 (SARS-CoV-2) is still spreading throughout the world and represents a major public health problem.

The first reports of this pandemic from Wuhan, China, revealed that up to 20% of hospitalized patients with COVID-19 developed bilateral interstitial pneumonia with hypoxemia (severe COVID-19), and nearly 5% progressed to acute respiratory distress syndrome (ARDS) requiring intensive care unit (ICU) admission and invasive mechanical ventilation (IMV) (critical COVID-19), potentially leading to multi-organ failure and death [1,2]. It was then shown that the progression to severe/critical COVID-19 was not primarily due to the direct damage induced by severe acute respiratory syndrome coronavirus 2 (SARS-CoV-2) itself but rather to an exaggerated systemic innate immune response to the virus. This aberrant immune response, comparable to other hyperinflammatory syndromes such as hemophagocytic lymphohistiocytosis and sepsis, is characterized by strikingly elevated levels of inflammatory cytokines (e.g., interleukin[IL]-1, IL-6) and C-reactive protein (CRP) [3]. The elevation of D-dimer and ferritin levels as well as moderate leukocytosis and lymphocytopenia are also frequent in severe/critical COVID-19 patients [2,4]. Eosinophils, whose absolute peripheral count is normally below 0.04 G/L, are usually normal or elevated in inflammatory lung diseases such as infections, asthma, or systemic vasculitis [5]; eosinopenia is much rarer, except in patients receiving specific treatments such as steroids, interferon alpha (IFNa), or anti-IL-5 therapies [5]. Whereas previous studies have suggested that eosinopenia may be present in patients with COVID-19, its clinical relevance and underlying mechanisms remain uncertain [6,7,8,9].

In this study, we explore the association between severe eosinopenia and a composite poor outcome, including the need for oxygen supplementation at >6 L/min, ICU admission, and in-hospital death, in a large cohort of COVID-19 patients hospitalized during the first and second COVID-19 waves in France. In addition, we investigate the potential mechanisms driving eosinopenia in COVID-19.

## 2. Materials and Methods

### 2.1. Patients and Outcomes

We retrospectively analyzed the records of COVID-19 patients hospitalized from March to June 2020 in three Marseille University hospitals, France. We included, in the study, all patients hospitalized for more than two days, excluding those who had received systemic glucocorticoid therapy in the last three months. In this cohort of patients, the standard of care did not include at the time the glucocorticoid therapy for severe COVID-19 patients as later recommended by the RECOVERY study [10]. Our study was approved by the institutional review board of the Assistance Publique-Hôpitaux de Marseille (RGPD2020-47). COVID-19 diagnosis was based on the presence of functional respiratory symptoms, typical radiological findings on lung computed tomography (CT) scans, and positive RT-PCR tests for SARS-CoV-2 using nasopharyngeal swabs. Clinical data (including demographic data, comorbidities, symptoms, CT scans, and clinical outcomes) were extracted from electronic medical records. Laboratory assessments upon admission consisted of complete blood count, T-cell subset phenotyping, inflammatory biomarkers (C-reactive protein [CRP], ferritin), and troponin, liver, and kidney tests. Circulating cortisol (electrochemiluminescence), interferon gamma-induced protein 10 (IP-10, also known as C-X-C motif chemokine ligand 10 [CXCL10], Luminex, Austin, TX, USA), IL-6 (Luminex, Austin, TX, USA), and soluble urokinase-type plasminogen activator receptor (suPAR, Virogates, Denmark) concentrations were prospectively measured in COVID-19 patients. Histopathological analyses were performed on lung specimens from 37 patients with fatal COVID-19 and severe eosinopenia from the “Hôpital Nord”, Marseille, France, and the “Santo Spirito” Hospital, Pescara, Italy.

**Figure 1:**
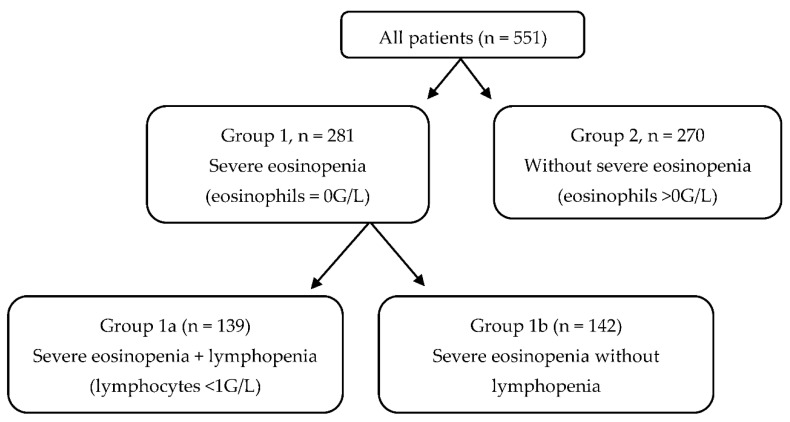
Flow Chart of the study cohort.

To investigate whether severe eosinopenia correlated with COVID-19 severity and prognosis, patients were divided into two groups according to their eosinophil levels on admission: patients with an absolute eosinophil count of 0 G/L were classified as “severe eosinopenia” group, and those with an eosinophil count greater than 0 G/L as “no severe eosinopenia” group. Furthermore, patients with severe eosinopenia were further stratified according to their lymphocyte levels (absolute lymphocyte count below or above 1 G/L), which have been previously shown to correlate with a COVID-19 worse outcome [11], corresponding to the groups “severe eosinopenia plus lymphocytopenia” and “severe eosinopenia without lymphocytopenia”. The groups are represented in the flow chart below (Figure 1). The occurrence of a composite poor outcome, including the need for oxygen therapy higher than 6 L/min, ICU admission, or in-hospital death, was assessed for all patients.

In addition, we compared the frequency of severe eosinopenia in COVID-19 patients with acute respiratory distress syndrome (ARDS) to that of non-COVID-19 patients with ARDS from a historical cohort from the “Hôpital Nord”, Marseille, France.

### 2.2. Statistical Analysis

Continuous variables with a normal distribution were described using the mean ± standard deviation (SD), and categorical variables were described using frequency with percentage. Univariate analyses were performed using chi^2^ or Fisher’s exact test for qualitative variables and t-test for quantitative variables. Multivariate logistic regression analysis was performed for “composite poor outcome” as the dependent variable. We firstly performed univariate logistic regression analyses, and we introduced into the multivariate model variables with a *p*-value < 0.20 in univariate analyses (after removing intermediary outcomes and collinear ones). We then applied a backward elimination so as to conserve variables whose adjusted *p*-value was less than 0.05. Age, sex, body mass index (BMI), hypertension, and diabetes were forced into the multivariate analysis.

Due to the high amount of missing data for BMI (168 patients did not have a reported BMI), and to check the robustness of the model with respect to the criterion “severe eosinopenia” in the multivariate analysis, a multiple imputation of the missing data for the BMI variable was performed. The variables used for multiple imputation of missing data were ferritin and presence of high blood pressure. We performed 20 samples with imputed data from which we performed the logistic regressions. The goodness-of-fit of the model was assessed using the Hosmer and Lemeshow test. First, we performed the multivariate analysis without BMI. Then, we performed multivariate analysis with multiple imputation of missing data for the BMI variable. Statistics were performed using SAS^®^ software. Comparisons of mean cortisol and IP-10 levels were performed using unpaired non-parametric t-test. Statistics were performed using the SAS^®^ software, and the statistical significance yielded at alpha = 0.05.

## 3. Results

A total of 551 COVID-19 patients were included in this study. On hospital admission, 73.50% (*n* = 405) of patients had eosinopenia (absolute eosinophil count <0.04 G/L), of whom 51% (*n* = 281) had undetectable eosinophils (severe eosinopenia). The main clinical, laboratory, and radiological characteristics of patients with severe eosinopenia are shown in Table 1 and compared to those of patients without severe eosinopenia.

Severe eosinopenia at admission was associated with a higher neutrophil-to-lymphocyte ratio (NLR) (7.66 vs. 6.07; *p* < 0.001), a lower lymphocyte count (*p* < 0.001) with a CD4+ lymphopenia (486/mm3 vs. 682/mm3; *p* = 0.001), and a higher platelet count (*p* < 0.001). Severe eosinopenia was also significantly associated with the elevation of inflammatory markers including CRP (*p* < 0.001), fibrinogen (*p* < 0.05), ferritin (*p* < 0.001), IP-10 (*p* < 0.05), and suPAR levels (*p* < 0.05). More precisely, the eosinophil count was negatively correlated with the suPAR level (r = 0.39, *p* < 0.01). Conversely, we found no difference in blood cortisol and IL-6 levels when comparing patients with and without severe eosinopenia (Table 2).

Severe eosinopenia on admission was associated with a composite poor outcome, (34.87% vs. 21.11%; *p* < 0.001, Table 3) and with death (15.30% vs. 7.03%, *p* < 0.001).

Specifically, severe eosinopenia was associated with a higher rate of in-hospital mortality (15.30% vs. 7.03%; *p* < 0.001), a longer requirement for oxygen therapy (12.26 days vs. 7.71 days; *p* < 0.001), and a higher length of hospital stay (mean 13.72 days vs. 11.81 days; *p* < 0.05). In addition, patients with severe eosinopenia showed more frequently severe or critical lung damage on their CT scans (60.5% vs. 50.7%, *p* < 0.05).

The concomitant presence of both severe eosinopenia and lymphocytopenia on admission was associated with even worse outcomes: patients with severe eosinopenia plus lymphopenia (*n* = 139) more frequently showed a composite poorer outcome (53.95% vs. 19.66%; *p* < 0.001), including a higher mortality rate (17.26% vs. 9.15%; *p* < 0.001) than other patients (Table 4).

Using multivariate analysis, severe eosinopenia, in general, was independently associated with a composite poor outcome (OR = 2.58 CI [1.77–3.75]; *p* < 0.0001), as were obesity (OR = 2.34 CI [1.5–3.66]; *p* < 0.001) and male gender (OR = 1.48 CI [1.02–2.15]; *p* < 0.05) (Table 5).

Severe eosinopenia in patients with COVID19-related ARDS was more frequent than in patients with non-COVID-19 ARDS (70% vs. 45%; *p* < 0.0001). Etiologies of non-COVID-19 ARDS are detailed in Table 6.

**Figure 2:**
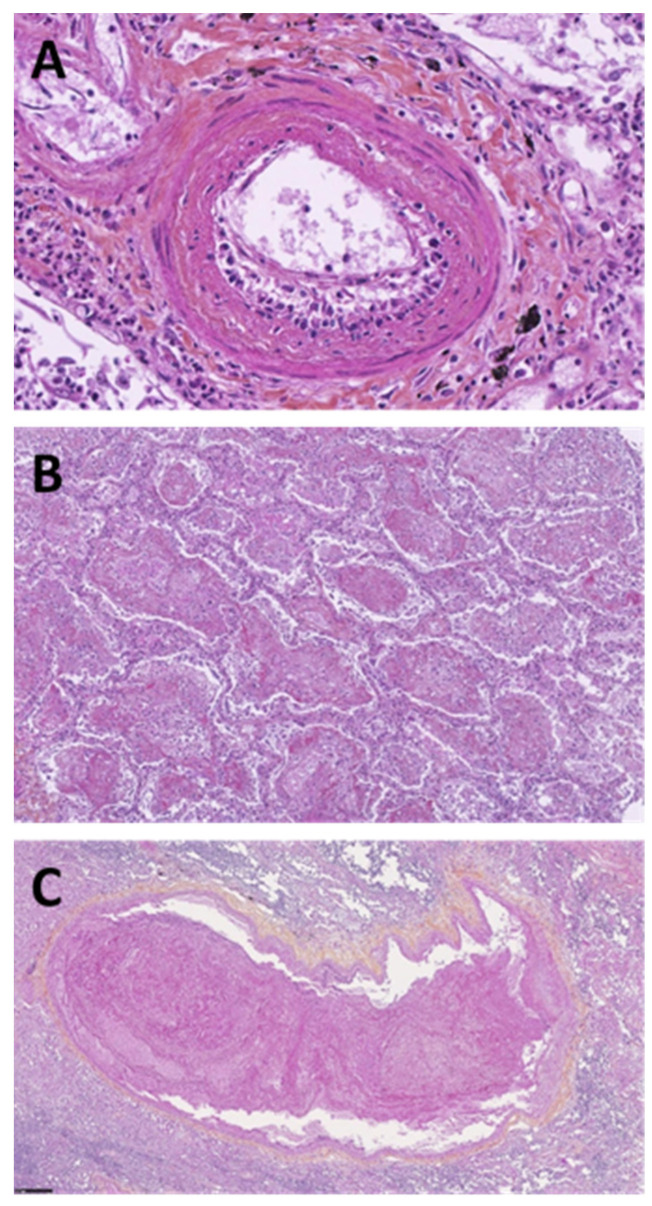
Lung histopathology in fatal COVID-19. (**A**) Hematein–eosin–saffron staining at magnification ×300; presence of endothelitis: thickening of the intima of a small-caliber arteriole with cell proliferation, intimal edema, and lymphocytic infiltrate. (**B**) Hematein–eosin–saffron staining at magnification ×70; filling of the alveoli with hyaline membranes and thick fibrin; presence of dystrophic pneumocytes desquamating in the lumens showing acute alveolar damage. (**C**) Hematein–eosin–saffron staining at magnification ×50; fibrin clot.

A histological analysis, performed in a series of 37 autopsies of patients who had died from COVID-19-related ARDS, revealed the presence of vascular wall thickening (100%), perivascular lymphocytic infiltrate (40%), thrombi (60%), small- and medium-sized vasculitis (80%), lung fibrosis (60%), diffuse interstitial disease (60%), alveolar condensation (60%), cell necrosis (40%), and bronchial destruction (80%), but in none of the analyzed sections was eosinophil trapping observed (Figure 2).

## 4. Discussion

In this multicentric cohort of 551 hospitalized COVID-19 patients, severe eosinopenia was significantly and independently associated with a composite poor outcome including the need for oxygen supplementation at >6 L/min, ICU admission, and in-hospital death. This confirms the previous findings. Indeed, in one of the first studies from Wuhan, China, reporting on the clinical characteristics of patients with COVID-19, eosinopenia was found in almost all patients who died (81.2%), whereas it was less frequent in patients who survived with non-severe and severe COVID-19 (60.7 and 47.6%, respectively) [12,13]. Similarly, in their study on the longitudinal hematologic variations associated with the progression of COVID-19 patients in China, Chen et al. found that most of the severe/critical and fatal patients demonstrated eosinopenia on admission. They also reported that eosinophils continually increased and reached significantly higher levels in survivors than in non-survivors [3]. More recently, in a smaller cohort of hospitalized COVID-19 patients, Tong et al. found that the death rate in a low eosinophils group was higher, and no patients died in the normal eosinophils group (16.7% vs. 0, *p* < 0.001) [14]. Altogether, we added evidence to the finding that the absolute eosinophil count may serve as a reliable prognostic biomarker for patients hospitalized with COVID-19.

In addition, eosinopenia has also been reported in patients with severe acute respiratory syndrome coronavirus (SARS-CoV) and Middle East respiratory syndrome (MERS-CoV) [15]. In patients with acute exacerbations of chronic obstructive pulmonary disease, lower eosinophil counts were associated with poorer clinical outcomes [16,17].

In this study, we found that the frequency of severe eosinopenia was significantly higher in patients with COVID-19-related ARDS than in those with COVID-19 unrelated ARDS. To avoid possible bias caused by the use of corticosteroids and its effect on eosinophils, we included only COVID-19 patients without recent or current exposure to the drug (and before the standard of care included glucocorticoids as recommended by the results of the RECOVERY study) [10]. Recently, Chen et al. found that higher eosinophil counts were related to lower 28-day mortality in a large cohort of ARDS patients (*n* = 2567) [18]. The authors showed that this relationship could be counteracted using corticosteroids.

However, the precise mechanisms underlying eosinopenia associated with COVID-19 remain unclear at this time. As eosinophils have anti-viral properties and the ability to migrate into tissues [5], the trapping of eosinophils into the injured lungs has been evoked to potentially account for the depletion of circulating eosinophils. In our analysis of lung histology from a series of 37 autopsies, we did not find evidence of eosinophilic pulmonary infiltrates in the lungs of patients with fatal COVID-19 and initial severe eosinopenia. This is consistent with previous histopathological studies [19].

In addition, severe eosinopenia may reflect the intense innate immune response associated with severe/critical COVID-19 and may thus represent an indicator of hyperinflammation/immune exhaustion. In this study, we observed that in COVID-19 patients, severe eosinopenia was associated with elevated circulating concentrations of IP-10, an inflammatory chemokine whose levels have been strongly associated with ARDS occurrence [20]. Furthermore, eosinopenia was associated with higher levels of suPAR, which derives from the cleavage of membrane-bound uPAR that occurs during intense immune activation [21]. Notably, suPAR has recently been demonstrated as an early inflammatory biomarker in patients with COVID-19 [22].

Stress-based cortisol responses which in other circumstances might lead to eosinopenia have been reported to be impaired in moderate-to-severe COVID-19 [23]. In this study, eosinopenia was not associated with higher plasma levels of cortisol, adding evidence to the fact that eosinopenia is not likely secondary to the cortisol inhibitory activity on eosinophil precursors in COVID-19 [24].

Despite the high number of patients included and the large data analyzed, our study has some limitations. First, its retrospective design is vulnerable to biases. Second, we analyzed the biological markers only at the time of hospital admission and not longitudinally. Third, we included patients hospitalized during the first and second waves in France, and as such our results cannot be extrapolated to other spatiotemporal populations. In particular, the strains circulating during these waves corresponded to clades 20A, 20B, 20C (first phase, February–May 2020), the Pangolin lineage B.1.177, and B.1.160 variant (second phase, June–December 2020) [25]; these are mostly different from those circulating during later waves and associated with different clinical outcomes [26].

Further studies—including in vitro, in vivo, and larger clinical studies—exploring the specific effects of a wider range of inflammatory cytokines in COVID-19 patients are needed. A possible direct cytotoxic effect of SARS-CoV-2 on eosinophils would merit further investigation. In addition, the distinct effects of different anti-cytokine strategies (e.g., corticosteroids, IL-1/IL-6/JAK-STAT inhibition) on eosinophils in patients with COVID-19 should be further appraised.

## 5. Conclusions

Severe eosinopenia can be a reliable biomarker associated with a composite poor outcome in hospitalized COVID-19 adult patients. It may reflect the magnitude of immune hyperactivation during severe-to-critical COVID-19.

## Figures and Tables

**Table 1:** Baseline characteristics and biological features of hospitalized COVID-19 patients with and without severe eosinopenia.

	Severe Eosinopenia (Group 1)	Without Severe Eosinopenia (Group 2)	*p*
	(*n* = 281)	(*n* = 270)	
Demographics: mean (standard deviation) or absolute number (percentage)			
Age, years	65.13 (15.02)	64.33 (16.32)	0.552
Male/female	131 (46.6%)/150 (53.4%)	139 (51.5%)/131 (48.5%)	0.253
Comorbidities: Absolute number (%)			
Diabetes	80 (28.47%)	78 (28.89%)	0.913
High blood pressure	121 (43.06%)	140 (51.85%)	0.042
Body mass index > 30 kg/m^2^	72 (25.62%)	53 (19.63%)	0.064
Cardiovascular disease *	37 (13.17%)	38 (14.07%)	0.756
Immunosuppression **	31 (11.03%)	39 (14.44%)	0.229
Chronic respiratory disease ***	43 (15.30%)	52 (19.26%)	0.219
Tobacco smoking	49 (17.44%)	71 (26.30%)	0.011
Initial laboratory results: mean (SD)			
Leucocytes, G/L	7.66 (5.83)	7.71 (4.65)	0.908
Neutrophils, G/L	6.05 (4.44)	5.38 (3.70)	0.05
Lymphocytes, G/L	1.02 (0.81)	1.53 (1.55)	<0.001
CD3 + (/mm3)	811 (522)	1075 (620)	0.021
CD3 + CD4 + (mm3)	485.8 (291.8)	682.2 (419.5)	0.001
CD3 + CD8 + (/mm3)	286.6 (237.6)	364.8 (256.5)	0.054
CD19 + (/mm3)	182.2 (193)	181.8 (159)	0.981
CD16 + CD56 + CD3- (/mm3)	196.1 (143.2)	211.9 (183.1)	0.543
Neutrophil to lymphocyte ratio	7.66 (13.95)	6.07 (6.38)	<0.001
Monocytes, G/L	0.51 (0.64)	0.96 (5.37)	0.169
Hemoglobin, g/dL	13.23 (1.94)	14.14 (10.95)	0.179
Platelets, G/L	198 (82)	240 (100)	<0.001
C-reactive protein, mg/L	112.3 (86.2)	82.9 (82.2)	<0.001
Ferritin, µg/L	1367 (1146)	890 (917)	<0.001
Fibrinogen, g/L	6.29 (1.57)	5.89 (1.70)	0.018
Albumin, g/L	36.74 (4.87)	36.70 (6.10)	0.945
Creatinin, µmol/L	92.86 (54.05)	94.17 (63.19)	0.794
ASAT, IU/L	61.51 (46.77)	45.21 (28.75)	0.003
LDH, IU/L	373.2 (129.5)	322.4 (130.4)	<0.001
Troponin, ng/L	42.34 (146.80)	30.69 (39.43)	0.4
CT scan: Absolute number (%)			
Severe or critical CT scan	170 (60.5%)	137 (50.7%)	0.033

* Coronary or peripheral vascular disease. ** Pre-existing cancer, immunosuppressant, HIV. *** Asthma or chronic obstructive pulmonary disease.

**Table 2:** suPAR, IP-10, IL-6, and cortisol levels in hospitalized COVID-19 patients with and without severe eosinopenia.

	Severe Eosinopenia (Group 1)	Without Severe Eosinopenia (Group 2)	*p*
	Mean (SD)		
suPAR	10.210 (5.4)	6.643 (6.6)	0.0334
IP-10	142.8 (122.9)	84.04 (70.2)	0.0301
Interleukin-6 (pg/mL)	47.37 (53.7)	51.35 (55.4)	0.7935
Blood cortisol (nmol/L)	458 (318)	483 (174)	0.7345

**Table 3:** Mortality and composite poor outcome including ICU admission and/or oxygen at >6 L/min and/or death in hospitalized COVID-19 patients with and without severe eosinopenia.

	Severe Eosinopenia (Group 1)	Without Severe Eosinopenia (Group 2)	*p*
	(*n* = 281)	(*n* = 270)	
	Absolute number (%)		
Composite poor outcome	98 (34.87%)	57 (21.11%)	<0.001
Death	43 (15.30%)	19 (7.03%)	<0.001

**Table 4:** Mortality and composite poor outcome including ICU admission and/or oxygen at >6 L/min and/or death in hospitalized COVID-19 patients with and without severe eosinopenia + lymphopenia.

	Severe Eosinopenia and Lymphopenia (Group 1a)	Other Patients	*p*
	(*n* = 139)	(*n* = 412)	
	Absolute number (%)		
Composite poor outcome	75 (53.95%)	81 (19.66%)	<0.001
Death	24 (17.26%)	38 (9.22%)	<0.001

**Table 5:** Multivariate analysis of factors associated with a composite poor outcome.

	OR (IC 95%)	*p*
Eosinophils = 0 G/L	2.58 (1.77–3.75)	<0.0001
Male gender	1.48 (1.02–2.15)	0.0371
BMI ≥ 30 kg/m^2^	2.34 (1.50–3.66)	0.0002
Age ≥ 65	1.35 (0.90–2.02)	0.1447
High blood pressure	1.36 (0.90–2.05)	0.1492
Type 2 diabetes	0.99 (0.64–1.51)	0.9465

**Table 6:** Etiology of ARDS not related to SARS-CoV2.

Etiology of ARDS Not Related to SARS-CoV2 (*n* = 118), Absolute Number (%)	
Bacterial pneumonia	67 (56.78%)
Ventilator-associated pneumonia	8 (6.78%)
Viral pneumonia	11 (9.32%)
Pulmonary embolism	3 (2.54%)
Pulmonary fibrosis	5 (4.24%)
Hemoptysis	4 (3.39%)
Atelectasis	6 (5.08%)
Drowning	1 (0.85%)
Traumatic	5 (4.24%)
Others	8 (6.78%)

## Data Availability

For original data, please contact the corresponding author.

## References

[1] Siddiqi H.K., Mehra M.R. (2020). COVID-19 Illness in Native and Immunosuppressed States: A Clinical-Therapeutic Staging Proposal. J. Heart Lung Transpl..

[2] Wang D., Hu B., Hu C., Zhu F., Liu X., Zhang J., Wang B., Xiang H., Cheng Z., Xiong Y. (2020). Clinical Characteristics of 138 Hospitalized Patients With 2019 Novel Coronavirus-Infected Pneumonia in Wuhan, China. JAMA.

[3] Chen G., Wu D., Guo W., Cao Y., Huang D., Wang H., Wang T., Zhang X., Chen H., Yu H. (2020). Clinical and immunological features of severe and moderate coronavirus disease 2019. J. Clin. Investig..

[4] Zhou F., Yu T., Du R., Fan G., Liu Y., Liu Z., Xiang J., Wang Y., Song B., Gu X. (2020). Clinical course and risk factors for mortality of adult inpatients with COVID-19 in Wuhan, China: A retrospective cohort study. Lancet.

[5] Lindsley A.W., Schwartz J.T., Rothenberg M.E. (2020). Eosinophil responses during COVID-19 infections and coronavirus vaccination. J. Allergy Clin. Immunol..

[6] Sun Y., Dong Y., Wang L., Xie H., Li B., Chang C., Wang F.-S. (2020). Characteristics and prognostic factors of disease severity in patients with COVID-19: The Beijing experience. J. Autoimmun..

[7] Cazzaniga M., Fumagalli L.A.M., D’angelo L., Cerino M., Bonfanti G., Fumagalli R.M., Schiavo G., Lorini C., Lainu E., Terragni S. (2021). Eosinopenia is a reliable marker of severe disease and unfavourable outcome in patients with COVID-19 pneumonia. Int. J. Clin. Pract..

[8] Soni M. (2021). Original Article: Evaluation of eosinopenia as a diagnostic and prognostic indicator in COVID-19 infection. Int. J. Lab. Hematol..

[9] Yan B., Yang J., Xie Y., Tang X. (2021). Relationship between blood eosinophil levels and COVID-19 mortality. World Allergy Organ J..

[10] Horby P., Lim W.S., Emberson J.R., Mafham M., Bell J.L., Linsell L., Staplin N., Brightling C., Ustianowski A., RECOVERY Collaborative Group (2021). Dexamethasone in Hospitalized Patients with Covid-19. N. Engl. J. Med..

[11] Lee J., Park S.-S., Kim T.Y., Lee D.-G., Kim D.-W. (2021). Lymphopenia as a Biological Predictor of Outcomes in COVID-19 Patients: A Nationwide Cohort Study. Cancers.

[12] Zhang J.-J., Dong X., Cao Y.-Y., Yuan Y.-D., Yang Y.-B., Yan Y.-Q., Akdis C.A., Gao Y.-D. (2020). Clinical characteristics of 140 patients infected with SARS-CoV-2 in Wuhan, China. Allergy.

[13] Du Y., Tu L., Zhu P., Mu M., Wang R., Yang P., Wang X., Hu C., Ping R., Hu P. (2020). Clinical Features of 85 Fatal Cases of COVID-19 from Wuhan. A Retrospective Observational Study. Am. J. Respir. Crit. Care Med..

[14] Tong X., Cheng A., Yuan X., Zhong X., Wang H., Zhou W., Xu X., Li Y. (2021). Characteristics of peripheral white blood cells in COVID-19 patients revealed by a retrospective cohort study. BMC Infect. Dis..

[15] Lee Y.-S., Chen C.-H., Chao A., Chen E.-S., Wei M.-L., Chen L.-K., Yang K.D., Lin M.-C., Wang Y.-H., Liu J.-W. (2005). Molecular signature of clinical severity in recovering patients with severe acute respiratory syndrome coronavirus (SARS-CoV). BMC Genom..

[16] Garnacho-Montero J., Huici-Moreno M.J., Gutiérrez-Pizarraya A., López I., Márquez-Vácaro J.A., Macher H., Guerrero J.M., Puppo-Moreno A. (2014). Prognostic and diagnostic value of eosinopenia, C-reactive protein, procalcitonin, and circulating cell-free DNA in critically ill patients admitted with suspicion of sepsis. Crit. Care.

[17] MacDonald M.I., Osadnik C.R., Bulfin L., Hamza K., Leong P., Wong A., King P.T., Bardin P.G. (2019). Low and High Blood Eosinophil Counts as Biomarkers in Hospitalized Acute Exacerbations of COPD. Chest.

[18] Chen H.-T., Xu J.-F., Huang X.-X., Zhou N.-Y., Wang Y.-K., Mao Y. (2021). Blood eosinophils and mortality in patients with acute respiratory distress syndrome: A propensity score matching analysis. World J. Emerg. Med..

[19] Xu Z., Shi L., Wang Y., Zhang J., Huang L., Zhang C., Liu S., Zhao P., Liu H., Zhu L. (2020). Pathological findings of COVID-19 associated with acute respiratory distress syndrome. Lancet Respir Med.

[20] Yang Y., Shen C., Li J., Yuan J., Wei J., Huang F., Wang F., Li G., Li Y., Xing L. (2020). Plasma IP-10 and MCP-3 levels are highly associated with disease severity and predict the progression of COVID-19. J. Allergy Clin. Immunol..

[21] Backes Y., van der Sluijs K.F., Mackie D.P., Tacke F., Koch A., Tenhunen J.J., Schultz M.J. (2012). Usefulness of suPAR as a biological marker in patients with systemic inflammation or infection: A systematic review. Intensive Care Med..

[22] Kyriazopoulou E., Poulakou G., Milionis H., Metallidis S., Adamis G., Tsiakos K., Fragkou A., Rapti A., Damoulari C., Fantoni M. (2021). Early treatment of COVID-19 with anakinra guided by soluble urokinase plasminogen receptor plasma levels: A double-blind, randomized controlled phase 3 trial. Nat. Med..

[23] Alzahrani A.S., Mukhtar N., Aljomaiah A., Aljamei H., Bakhsh A., Alsudani N., Elsayed T., Alrashidi N., Fadel R., Alqahtani E. (2021). The Impact of COVID-19 Viral Infection on the Hypothalamic-Pituitary-Adrenal Axis. Endocr. Pract..

[24] Bro-Rasmussen F. (1973). Effect of cortisol on the eosinophils in the rat spleen. Autoradiographic studies. Scand. J. Haematol..

[25] Hoang V.-T., Colson P., Levasseur A., Delerce J., Lagier J.-C., Parola P., Million M., Fournier P.-E., Raoult D., Gautret P. (2021). Clinical outcomes in patients infected with different SARS-CoV-2 variants at one hospital during three phases of the COVID-19 epidemic in Marseille, France. Infect. Genet. Evol..

[26] Dao T.L., Hoang V.T., Nguyen N.N., Delerce J., Chaudet H., Levasseur A., Lagier J.C., Raoult D., Colson P., Gautret P. (2021). Clinical outcomes in COVID-19 patients infected with different SARS-CoV-2 variants in Marseille, France. Clin. Microbiol. Infect..

